# Spasm of the Colon

**Published:** 1831

**Authors:** 


### XXXVI.

### Spasm of the Colon.

Mr. Lyon has published some observations on this subject, in the April number of our contemporary, the London Med. and Surg. Journal. The complaint, Mr. L. observes is more particularly incidental to females from their inattention to their bowels. The symptoms are these. After constipation, there are paroxysms of pain, rising into violent spasms, with remissions, but not complete intermissions. The pain is referred to the transverse arch and sigmoid flexure of the colon. The abdomen is flaccid—pressure relieves the pain—and nausea is sometimes present.

The tongue is usually moist and white, the pulse rarely beyond 85, full, and compressible. There is little thirst, or increase of temperature on the surface. After a day or two, a purging comes on, and the disease is commonly treated as diarrhœa, though the cause, our author thinks, is scybala. The treatment which he has found most successful, consists in the administration of purgatives combined with opiates—for instance, a black draught with eight minims of laudanum every four hours till the stools put on a healthy character.

---

